# A randomized, double-blind, placebo-controlled, proof-of-concept trial of creatine monohydrate as adjunctive treatment for bipolar depression

**DOI:** 10.1007/s00702-017-1817-5

**Published:** 2017-11-24

**Authors:** Ricardo Alexandre Toniolo, Michelle Silva, Francy de Brito Ferreira Fernandes, José Antonio de Mello Siqueira Amaral, Rodrigo da Silva Dias, Beny Lafer

**Affiliations:** Bipolar Disorder Program (PROMAN), Instituto de Psiquiatria, Hospital das Clinicas HCFMUSP, Faculdade de Medicina, Universidade de Sao Paulo, Rua Dr. Ovídio Pires de Campos, 785, São Paulo, SP 05403-010 Brazil

**Keywords:** Adjunctive therapy, Creatine monohydrate, Randomized controlled trial, Double-blind method, Bipolar disorder, Depression

## Abstract

Depressive episodes are a major cause of morbidity and dysfunction in individuals suffering from bipolar disorder. Currently available treatments for this condition have limited efficacy and new therapeutic options are needed. Extensive research in the pathophysiology of bipolar disorder points to the existence of mitochondrial and bioenergetic dysfunction. We hypothesized that creatine monohydrate, a nutraceutical that works as a mitochondrial modulator, would be effective as an adjunctive therapy for bipolar depression. We conducted a double-blind trial in which 35 patients with bipolar disorder type I or II in a depressive episode by DSM-IV criteria and in use of regular medication for the treatment of this phase of the disease were randomly allocated into two adjunctive treatment groups for 6 weeks: creatine monohydrate 6 g daily (*N* = 17) or placebo (*N* = 18). Primary efficacy was assessed by the change in the Montgomery–Åsberg Depression Rating Scale (MADRS). We did not find a statistically significant difference in the comparison between groups for the change in score on the MADRS after 6 weeks in an intention-to-treat (ITT) analysis (*p* = 0.560; Cohen’s *d* = 0.231). However, we found significant superiority of creatine add-on vs. placebo when we considered the remission criterion of a MADRS score ≤ 12 at week 6 analyzing the outcome of the 35 randomized patients on ITT (52.9% remission in the creatine group vs. 11.1% remission in the placebo group) and of the 23 completers (66.7% remission in the creatine group vs. 18.2% remission in the placebo group) (*p* = 0.012; OR = 9.0 and *p* = 0.036; OR = 9.0, respectively). Two patients who received creatine switched to hypomania/mania early in the trial. No clinically relevant physical side-effects were reported or observed. This proof-of-concept study, aiming to restore brain bioenergetics using an adjunctive mitochondrial modulator, is not conclusive on the efficacy of creatine add-on for bipolar depression, but suggests that this compound may have a role in the adjunctive treatment of this phase of the illness. Further investigation through randomized controlled trials with larger samples should be conducted to verify the efficacy of creatine supplementation for bipolar depression and also for subsyndromal depressive symptoms.

## Introduction

Studies have revealed that the greatest burden in bipolar disorder is caused by the depressive episodes (Judd et al. 2002). Data from the Stanley Foundation Network indicated that 45% of patients with bipolar depression that were treated over a year with various pharmacological regimens which are commonly used in clinical practice did not improve or even worsened (Post et al. 2010).

Multiple lines of evidence strongly implicate mitochondrial and bioenergetic dysfunction in the pathophysiology of bipolar disorder (Morris et al. 2017; Kato 2016; Scaini et al. 2016; Nierenberg et al. 2013; Clay et al. 2011). A study using phosphorus magnetic resonance spectroscopy (^31^P-MRS) showed that patients with bipolar depression had significant reductions in the levels of phosphocreatine (PCr) in the left frontal lobe. Furthermore, PCr levels were negatively correlated with the severity of depressive symptoms (Kato et al. 1995). Other studies with ^31^P-MRS reported a reduction in PCr levels in the whole brain of adults with bipolar disorder type II (Kato et al. 1994) and in gray matter in adolescents with bipolar disorder type I in manic or euthymic states, as well as a decrease in the levels of ATP in white matter (Dudley et al. 2016). Moreover, patients with bipolar disorder presented deficiency in the availability of ATP from PCR through the creatine kinase reaction in occipital lobe during visual stimulation as a paradigm of increase in energy demands (Yuksel et al. 2015).

Some proof-of-concept clinical trials have found interesting antidepressive effects of mitochondrial modulators on bipolar disorder. A double-blind, placebo-controlled study showed that adjunctive *N*-acetylcysteine improved functioning and depressive symptoms in patients with bipolar disorder after 24 weeks (Berk et al. 2008). Open trials showed that adjunctive treatment with triacetyluridine may decrease depressive symptoms in bipolar disorder (Jensen et al. 2008) and that adjunctive Coenzyme Q10 was associated with a significant reduction in total MADRS score in geriatric bipolar depression (Forester et al. 2015).

We hypothesized that the mitochondrial modulator creatine monohydrate might have a role as an adjuvant treatment for bipolar depression. Creatine is a non-essential dietary component found primarily in meat and fish and endogenously produced by the liver, kidneys and pancreas (Wyss and Kaddurah-Daouk 2000). Oral intake of creatine monohydrate can increase brain concentrations of creatine and phosphocreatine in humans (Dechent et al. 1999; Lyoo et al. 2003). This suggests that supplementation could mitigate bioenergetic abnormalities by providing additional substrate to produce ATP. In fact, a double-blind placebo-controlled trial that included fifty-two female subjects with unipolar depression showed that supplementation with creatine monohydrate (5 g daily) for 8 weeks as an augmentation of treatment with escitalopram was safe and effective (Lyoo et al. 2012). A subsample of 34 patients that were enrolled in this trial also participated in multimodal neuroimaging assessments and those who had received creatine supplementation had levels of *N*-acetyl aspartate (NAA) increased in the prefrontal cortex and rich club connections enriched as evidenced through proton magnetic resonance spectroscopy (^1^H-MRS) and diffusion tensor imaging (DTI), respectively (Yoon et al. 2016).

Increased oxidative stress has been extensively implicated in the pathophysiology of bipolar disorder. A meta-analysis revealed that lipid peroxidation, nitric oxide (NO) activity and oxidative damage to DNA and RNA are all increased in patients with bipolar disorder (Brown et al. 2014). Creatine has shown itself capable of removing superoxide and peroxynitrite with a direct antioxidant power even greater than that of glutathione (Lawler et al. 2002; Sestili et al. 2006). Furthermore, creatine seems to have neuromodulatory activity (Royes et al. 2008) and anti-immobilizing effect on the caudal suspension test in animal models, which is a paradigm of antidepressant activity in preclinical models (Cunha et al. 2015).

The varied effects of creatine led us to expect that supplementation with this nutraceutical would be associated with improvement in depressive symptoms when used as an adjunctive treatment for bipolar depression in a proof-of-concept 6-week, randomized, double-blind, placebo-controlled clinical trial.

## Materials and methods

### Setting

The study protocol received approval from the local institutional review board and was recorded in the public database clinicaltrials.gov (NCT 01655030) before beginning to recruit subjects. All the volunteers signed a fully explained written informed consent form prior to their inclusion in the trial. The study procedures are in accordance with the Helsinki Declaration of 1975 and patients were given easy access to prompt psychiatric reevaluation during the whole period of the trial in case of clinical need.

Medical consultations and collection of blood samples were carried out in the Bipolar Disorder Research Program (PROMAN) outpatient clinic at the Instituto de Psiquiatria, Hospital das Clinicas HCFMUSP, Faculdade de Medicina, Universidade de Sao Paulo. Laboratory tests were performed at the Central Laboratory of the Hospital das Clinicas.

### Participants

Subjects aged 18–59 years who met DSM-IV (Diagnostic and Statistical Manual of Mental Disorders, Fourth Edition) criteria for bipolar disorder type I or II currently depressed as assessed using the Structural Clinical Interview for DSM-IV Axis I Disorders—Patient version (First et al. 1996) and whose score on the Montgomery–Åsberg Depression Rating Scale (MADRS) (Montgomery and Åsberg 1979) was 20 or greater were recruited to the study. The exclusion criteria included alcohol or other substance abuse in the past 2 weeks or meeting dependence criteria in the past 2 months, a diagnosis of dementia, delirium, epilepsy or mental retardation, clinically unstable medical conditions and a history of hypersensitivity to creatine. Patients clinically considered as at high risk for suicidal, homicidal or automutilatory behavior and pregnant or lactating women were not included. Patients who were taking antipsychotics or mood stabilizers were included in the trial if the dosages had been stable for at least 2 weeks; those who were on antidepressants were included if the dosages had been stable for at least 4 weeks.

### Procedures

After a primary screening via email or phone call, volunteers were invited to attend to a medical visit at our outpatients’ facilities. They were instructed to attend to this screening accompanied by a family member and their medical records were analyzed when available.

Subjects who were found to be not eligible to the study after the medical screening were referred for follow-up treatment as usual. Those who met the inclusion criteria and signed a consent form continued in the Structured Clinical Interview for DSM-IV Axis I Disorders (SCID) conducted by the psychiatrist. At the end of the interview, the patient was scheduled to the baseline visit within a few days, when subjects were then allocated in one of the treatment groups after a reevaluation by the same psychiatrist to check whether the patient still met the inclusion criteria.

Patients included in the trial were randomly assigned in a 1:1 ratio to receive supplementation with creatine monohydrate (creatine group) or placebo (placebo group) in random permuted blocks of four. The allocation sequence was generated by the coordinator pharmacist in the Pharmacy Division of Hospital das Clinicas, Faculdade de Medicina, Universidade de Sao Paulo through a web platform (http://www.graphpad.com). The pharmacist was not involved in any other phase of the research. The research assistant, the outpatient clinic’s nurse, the psychiatrist and the patients were all blind regarding allocation concealment.

At each biweekly visit, patients received from the research assistant a package with 14 laminated and sealed sachets containing 6 g of creatine monohydrate soluble powder (creatine group) or 6 g of corn starch (placebo). They were prescribed to ingest the content of one sachet daily diluted in the drink they preferred, at lunchtime. Soluble creatine monohydrate (Creapure^®^, AlzhChem Troserg GmbH Germany, US Patent #5,719,319) consists of a white, tasteless and odorless powder. Patients remained along the 6-week trial using the same drug regimens that were current at baseline. Three changes were exceptionally allowed due to insomnia.

Patients were evaluated by the same psychiatrist (R.A.T.) in all the visits. The baseline included a complete medical and psychiatric history, physical examination (including weight, height and blood pressure measurements) and psychometric assessment through the MADRS, the 17-item Hamilton Depression Rating Score (HAMD) (Hamilton 1967), the Young Mania Rating Scale (YMRS) (Young et al. 1978), the Clinical Global Impressions scale (CGI) (Guy 1976) and the Functioning Assessment Short Test (FAST) (Rosa et al. 2007). The psychometric instruments were readministered at each visit, except for FAST that was rechecked only at end point. A questionnaire covering the events that were most commonly reported in previous studies of creatine supplementation was applied at each visit. At each reassessment visit, the sachets that were not consumed in the previous 14 days were returned to the psychiatrist and accounted for at the end of the 6-week trial as a measure of adherence to protocol. Patients who consumed less than 11 of 14 sachets distributed at each visit were considered non-adherents and excluded from the study. Blood was collected between 8 and 10 a.m. at baseline and end point for routine laboratory tests as safety measures.

### Outcome measures

The primary outcome measure was change in MADRS after 6 weeks when compared to baseline. Secondary outcome measures consisted in the changes of the scores on the HAM-D, CGI-Severity and FAST and response and remission rates in the same period. We defined two steps of response: reduction of 25% or more (partial response) and reduction of 50% or more (complete response) using the MADRS score. Remission was defined as a MADRS score of 12 or less, as it has been defined in important published trials in the field (Calabrese et al. 2005; Loebel et al. 2014; McElroy et al. 2010; Young et al. 2010).

### Statistical analysis

After verifying the normality assumptions with the Kolmogorov–Smirnov test, we used the Chi-square test for categorical variables and the Student’s *t* test for continuous variables to compare the treatment groups regarding demographic and clinical variables. Intention-to-treat (ITT) analyses were performed with the last observation carried forward (LOCF) method. Odds ratios were calculated as measures of effect sizes of the statistically significant findings.

In this study, we considered as significant an *α* level of 0.05 for two-tailed tests. Data were analyzed with IBM SPSS software, version 23.0.

## Results

**Fig. 1:**
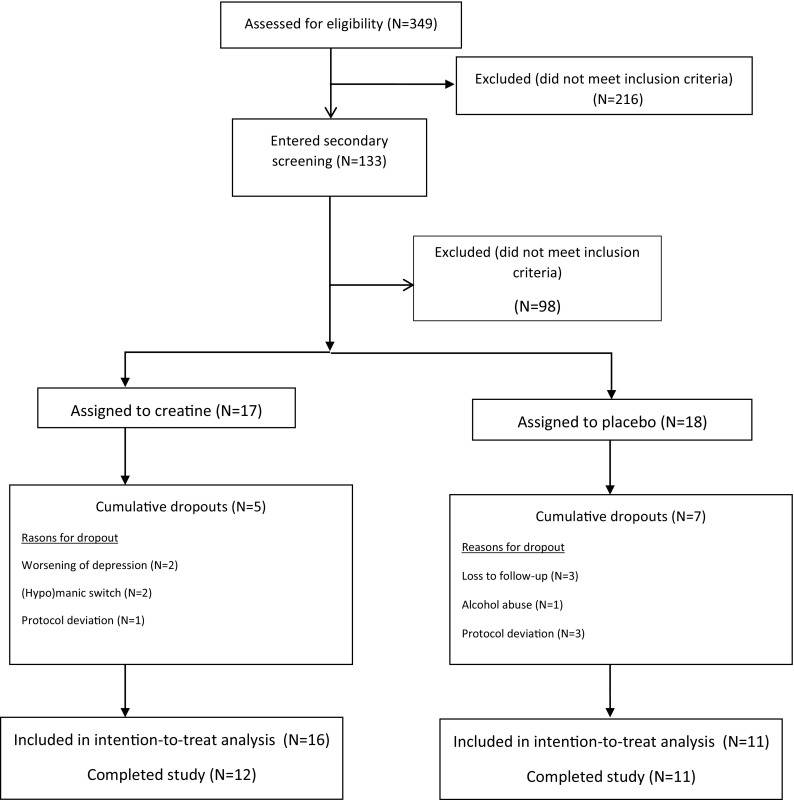
Screening, randomization and disposition of 35 patients with bipolar depression assigned to adjunctive treatment with creatine monohydrate or placebo

Thirty-five subjects were included in the trial and randomly allocated to receive creatine monohydrate (*N* = 17; 48.6%) or placebo (*N* = 18; 51.4%) (Fig. 1).

Twenty-three patients completed the study. There was no significant difference in dropout rates between the treatment groups: 29.4% in the creatine group (*N* = 5) and 38.9% in the placebo group (*N* = 7) (*χ* ^2^ = 0.054, *df* = 1, *p* = 0.815). We lost one patient in the placebo group to follow-up prior to the first reassessment due to reemployment, which disabled him to attend the study visits. Two subjects in the placebo group were lost to follow-up before any reassessment and we could no longer contact them. Three patients allocated in the placebo group deviated from protocol (they changed medications or their dosages without the consent of the psychiatrist) and were excluded from the study before the first reevaluation; the same happened to a patient in the creatine group soon after week 2. One patient in the creatine group presented worsening of depression and suicidal ideation at week 4 and was then excluded. Another patient in the creatine group who had a worsening of depressive symptoms at week 2 and attempted suicide was also excluded from the trial. One patient in the creatine group switched to hypomania at the end of week 2 and another from the same group switched to mania at the beginning of week 2 and were excluded. We also excluded a patient in the placebo group before week 2 due to heavy alcohol abuse in the first 10 days of the trial.

Two changes in prescription throughout the study were made in the creatine group (12.5%) and one in the placebo group (9.1%), all due to insomnia (*p* = 1.000; Fisher’s exact test). For one patient in the creatine group we prescribed zolpidem 10 mg q.h.s. and for another of the same group, zopiclone 7.5 mg q.h.s. A patient in the placebo group had an increase in the prescribed dosage of nitrazepam from 5 to 10 mg q.h.s. Adherence to study medication was very high in both groups (96.7% in the creatine group and 99.5% in the placebo group; *p* = 0.117).

**Fig. 2:**
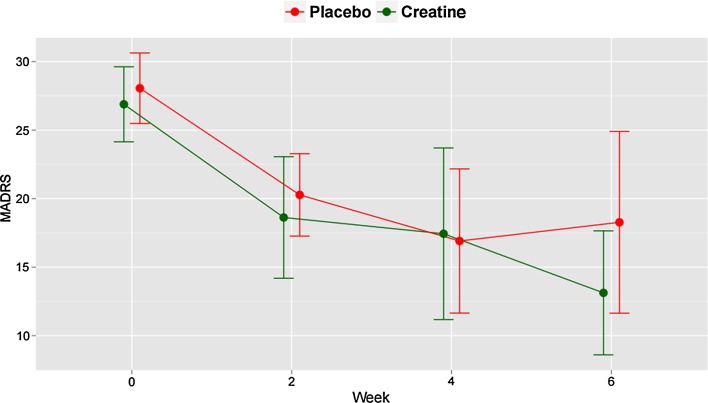
Observed and last observation carried forward Montgomery–Åsberg Depression Rating Scale (MADRS) scores at each treatment week for 27 patients with bipolar depression assigned to a 6-week adjunctive treatment with creatine monohydrate or placebo

An a priori decision was made to only include subjects with at least one postbaseline assessment in a primary ITT analysis using the last observation carried forward (LOCF) method (*N* = 16 in the creatine group; *N* = 11 in placebo group). The demographic (sex, age, education, marital status, occupation) and clinical (type and age of onset of BD, history of psychiatric hospitalization, lifetime psychotic symptoms, lifetime anxiety disorders, lifetime alcohol or other substances abuse or dependence, current use of different classes of psychotropics, scores on psychometric rating scales, BMI) at baseline of patients who received at least one reevaluation were similar in both groups, with the exception of scores on YMRS and FAST which were higher in the placebo group (*p* = 0.046 and 0.010, respectively) (Table 1). We did not find statistically significant differences between the groups in the comparison of the changes in scores on the MADRS (*p* = 0.560; *t* test; Cohen’s *d* = 0.231) (Table 2), HAM-D (*p* = 0.345; *t* test), YMRS (*p* = 0.125; *t* test), CGI-S (*p* = 0.317; *t* test) or FAST (*p* = 0.615; *t* test) and in rates of response or remission after 6 weeks. Figure 2 shows the changes in the scores on MADRS as LOCF.

**Table 1:** Baseline characteristics of 27 depressed patients with bipolar disorder (BD) randomly assigned to adjunctive treatment with creatine monohydrate or placebo

Characteristic	Creatine (*N* = 16)	Placebo (*N* = 11)	*p*
Demographic variables			
Female sex, *N* (%)	12 (75.0%)	8 (72.7%)	1.000^c^
Age in years, mean (SD)	44.5 (8.4)	43.0 (10.2)	0.679^b^
Education in years, mean (SD)	13.4 (2.34)	11.9 (3.67)	0.254^b^
Marital status			0.648^a^
Married, *N* (%)	5 (31.3%)	5 (45.5%)	
Single, *N* (%)	7 (43.8%)	4 (36.4%)	
Consensual marriage, *N* (%)	2 (12.5%)	1 (9.1%)	
Divorced, *N* (%)	1 (6.3%)	0 (0.0%)	
Separated, *N* (%)	0 (0.0%)	1 (9.1%)	
Widowed, *N* (%)	1 (6.3%)	0 (0.0%)	
Occupational status			0.509^a^
Employed, *N* (%)	7 (43.8%)	2 (18.2%)	
Out of job market, *N* (%)	1 (6.2%)	1 (9.1%)	
Unemployed—job seeking, *N* (%)	6 (37.5%)	6 (54.5%)	
Employed, on sick leave, *N* (%)	2 (12.5%)	1 (9.1%)	
Long-term disability, *N* (%)	0 (0.0%)	1 (9.1%)	
Clinical variables			
Type of BD			1.000^c^
I, *N* (%)	10 (62.5%)	7 (63.6%)	
II, *N* (%)	6 (37.5%)	4 (36.4%)	
Age of onset of BD in years, mean (SD)	23.4 (11.4)	20.8 (9.0)	0.586^b^
Lifetime psychotic symptoms, *N* (%)	11 (68.8%)	6 (54.5%)	0.687^c^
History of psychiatric hospitalization, *N* (%)	9 (56.2%)	3 (27.3%)	0.239^c^
Current medication			
Lithium, *N* (%)	3 (18.8%)	3 (27.3%)	0.662^c^
Anticonvulsivant, *N* (%)	12 (75.0%)	10 (90.9%)	0.618^c^
Atypical antipsychotic, *N* (%)	10 (62.5%)	6 (54.5%)	0.710^c^
ISRS/ISRSN antidepressant, *N* (%)	3 (18.8%)	4 (36.4%)	0.391^c^
Other antidepressant, *N* (%)	2 (12.5%)	1 (9.1%)	1.000^c^
Benzodiazepine/hypnotic, *N* (%)	5 (31.3%)	5 (45.5%)	0.687^c^
Lifetime psychiatric comorbidity			
Alcohol abuse/dependence, *N* (%)	1 (6.2%)	2 (18.2%)	0.549^c^
Non-alcoholic substance abuse/dependence, *N* (%)	1 (6.2%)	0 (0.0%)	1.000^c^
Anxiety disorder, *N* (%)	5 (31.3%)	10 (90.9%)	0.075^a^
Clinical rating scores			
MADRS, mean (SD)	27.1 (5.4)	30.1 (5.2)	0.167^b^
HAM-D, mean (SD)	15.1 (4.3)	17.8 (3.5)	0.103^b^
YMRS, mean (SD)	1.31 (1.40)	2.55 (1.64)	0.046^b^
CGI-S, mean (SD)	4.38 (0.50)	4.27 (0.47)	0.567^b^
FAST, mean (SD)	34.0 (14.7)	50.2 (14.3)	0.010^b^
Body mass index, mean (SD)	28.0 (4.3)	28.3 (5.3)	0.851^b^

^a^Calculated by Chi-square analysis

^b^Calculated by Student’s *t* test

^c^Calculated by Fisher’s exact test

**Table 2:** Baseline and end point scores in psychometric assessment scales for 27 patients with bipolar depression assigned to a 6-week adjunctive treatment with creatine monohydrate or placebo according to last observation carried forward

Score	Creatine		Placebo		*t*	*p* ^a^
	Baseline (*N* = 16) Mean (SD)	End point (*N* = 16) Mean (SD)	Baseline (*N* = 11) Mean (SD)	End point (*N* = 11) Mean (SD)		
MADRS	27.1 (5.40)	13.1 (8.49)	30.1 (5.19)	18.3 (9.87)	− 0.591	0.560
HAM-D	15.1 (4.27)	7.87 (4.96)	17.8 (3.55)	12.3 (4.69)	− 0.964	0.345
YMRS	1.31 (1.40)	4.00 (6.29)	2.55 (1.64)	1.82 (2.75)	1.588	0.125
CGI-S	4.38 (0.50)	2.94 (0.93)	4.27 (0.47)	3.27 (1.19)	− 1.021	0.317
FAST	34.0 (14.7)	19.2 (16.2)	50.2 (14.3)	29.7 (18.9)	0.512	0.615

^a^Calculated by Student’s *t* test

We performed an ITT analyses considering the 35 patients ‘as randomized’ for the discrete outcomes and found statistically significant differences between the groups for the rates of partial response (82.4% in the creatine group vs. 38.4% in the placebo group; *p* = 0.015, OR = 7.3; Fisher’s exact test) and remission (52.9% in the creatine group vs. 11.1% in the placebo group) (*p* = 0.012; OR = 9.0; Fisher’s exact test). Rates of complete response did not differ between the groups (*p* = 0.164; Fisher’s exact test).

Sex-specific antidepressant effects of creatine have been suggested by preclinical data that indicate antidepressant activity specifically in females (Allen et al. 2010). In this context, a post hoc intent-to-treat analysis was conducted comparing the changes in the scores on MADRS between the treatment groups after 6 weeks considering only the female patients (*N* = 12 in the creatine group; *N* = 8 in the placebo group) who received at least one postbaseline assessment, and found no statistically significant difference (*p* = 0.814; *t* test).

A secondary per-protocol analysis was carried out considering only the patients who completed the 6-week trial (*N* = 12 in the creatine group; *N* = 11 in the placebo group). We did not find statistically significant differences between the groups of completers on the changes on MADRS (*p* = 0.435; *t* test) and on the rates of total and partial responses. Six patients in the creatine group (50.0%) and four in the placebo group (36.4%) showed a reduction of at least 50% on the score on MADRS after 6 weeks (*p* = 0.680; Fisher’s exact test). Eleven patients in the creatine group (91.7%) and 7 (63.6%) in the placebo group had a reduction of at least 25% on the score on MADRS (*p* = 0.155; Fisher’s exact test). We found statistically significant superiority of creatine supplementation in the completers on the rates of remission: eight patients (66.7%) remitted in the creatine group and two in the placebo group (18.2%) (*p* = 0.036; OR = 9.0; Fisher’s exact test).

All the reported or observed adverse events during the clinical trial were mild, transient and improved or remitted without specific interventions and resulted in little or no clinical impact. No patient exhibited serum BUN or creatinine levels above the upper limits of normality both at baseline and after 6 weeks of treatment (Table 3).

**Table 3:** Adverse events reported by subjects with bipolar depression during a 6-week adjunctive treatment trial with creatine monohydrate or placebo

Adverse event	Creatine (*N* = 16)	Placebo (*N* = 11)	*p* ^a^
	*N* (%)	*N* (%)	
Pruritus	1 (6.3)	2 (18.2)	0.548
Cramps	1 (6.3)	1 (9.1)	1.000
Headache	2 (12.5)	6 (54.5)	0.033
Constipation	3 (18.8)	3 (27.3)	0.662
Diarrhea	2 (12.5)	1 (9.1)	1.000
Limb edema	2 (12.5)	2 (18.2)	1.000
Nausea	0 (0.0)	3 (27.3)	0.056
Vomiting	0 (0.0)	1 (9.1)	0.407
Reflux	1 (6.3)	2 (18.2)	0.549
Somnolence	1 (6.3)	3 (27.3)	0.273
Dizziness/vertigo	2 (12.5)	2 (18.2)	1.000
Asthenia	0 (0.0)	3 (27.3)	0.056
Abdominal pain	1 (6.3)	3 (27.3)	0.273
Other	1 (6.3)^b^	1 (9.1)^c^	1,000

^a^Calculated by Fisher’s exact test

^b^Heartburn

^c^Increased sweating

## Discussion

We believe that the current study is, to our knowledge, the first randomized, double-blind, placebo-controlled clinical trial that evaluated the efficacy, safety and tolerability of creatine monohydrate as adjunctive therapy for bipolar depression.

Patients included in this study presented a depressive episode of moderate severity according to the MADRS score (mean = 27.1, SD = 5.4 in the creatine group; mean = 30.1, SD = 5.2 in the placebo group). These average scores on the MADRS at baseline were very like the ones that were verified in the sample (*N* = 52) of subjects with unipolar depression that was studied by Lyoo et al. in an 8-week randomized, double-blind, placebo-controlled trial of creatine monohydrate (5 g/day) as an augmentation to the treatment with escitalopram and in which they found improved response in the creatine group as early as week 2 according to the scores on HAMD, MADRS and CGI (Lyoo et al. 2012).

We found complete response rates after 6 weeks of 50% in the creatine group, which is close to the pooled response rate (55.1%) found from 17 randomized, double-blind, placebo-controlled trials (*N* = 6578) that studied the efficacy for bipolar depression of drugs currently used for the treatment of this condition (Iovieno et al. 2016). We also detected more pronounced although non-statistically significant reductions in scores on the MADRS in the group that received creatine (reduction of 14 points in the creatine group vs. 11.8 points in the placebo group on ITT analysis; reduction of 15 points in the creatine group vs. 11.8 in the placebo group for the completers). We chose change in MADRS score as the primary outcome measure, unlike Lyoo et al. who chose change in HAMD in their study in unipolar depression. The MADRS scale was originally designed to remedy some of the psychometric limitations of HAMD and to be more sensitive to changes in score during treatment. It focuses on core symptoms of the depressive syndrome, rather than somatic and psychomotor symptoms which are emphasized in HAMD and distinguishes more accurately between unipolar and bipolar depression type I (Carneiro et al. 2015). In our study, the psychometric scales were applied for all participants throughout the 6-week trial by the same psychiatrist (R.A.T).

Our sample included men and women, with a female predominance (75.0% in the creatine group; 72.7% in the placebo group). The sample of patients with unipolar depression studied by Lyoo et al. included only women, similar to the one studied by Kondo et al. which included in an open trial only female teenagers with unipolar depression who had been using fluoxetine for at least 8 weeks. They received creatine monohydrate (4 g/day) for 8 weeks and had a significant increase in PCR levels as measured by ^31^P-MRS and a decrease of 56% in the score on the Children’s Depression Rating Scale—Revised. It is speculated that the cyclical variations in ovarian hormones, particularly estradiol, stimulate the activity of creatine kinase and amplify the rate at which ATP is restored from PCR in neurons, enhancing the antidepressant effects of creatine in women (Kondo et al. 2011). In this context, Renshaw et al. observed that baseline levels of high energy phosphates in the brain predicted a subsequent response to fluoxetine in women with unipolar depression, but not in men. Our study did not find a significant improvement of depressive symptoms in the subsample of female participants.

A large percentage of patients in the study by Lyoo et al. had never taken psychotropic medications, and volunteers should have been at least 8 weeks without using any psychotropic drug to be included in the trial. In our study, patients were already using commonly prescribed medications for the treatment of bipolar depression in clinical practice, to which they had not responded. Therefore, our sample of bipolar-depressed patients might present a more treatment-resistant depressive syndrome. Moreover, although there is considerable evidence that mitochondrial and bioenergetic dysfunctions play a key role in the pathophysiology of unipolar depression (Gardner and Boles 2011), these mechanisms may not exactly coincide with the ones implicated in bipolar disorder, so that we cannot simply extrapolate from the data on the efficacy of mitochondrial modulators for unipolar depression to samples of patients with bipolar depression.

We found a numerical increase on the score on YMRS in the creatine group (from 1.3 to 4.0) and one could argue that in a large sample, this result may turn out to be statistically relevant when compared to the decrease on YMRS in the placebo group, but we should notice that the mean score of 4.0 corresponds to a status of remission on YMRS according to the International Society for Bipolar Disorders Task Force (Tohen et al. 2009). Two patients in the creatine group were excluded from the study during the first 2 weeks due to hypomanic and manic switches; both were taking lithium and quetiapine. While nothing can be asserted regarding a possible causal role of creatine in these events, we could reasonably expect that boosting mitochondrial energy generation may trigger manic symptoms, as we hypothesize that bipolar disorder may consist of a biphasic disorder of energy generation, increased in mania and decreased in depression (Morris et al. 2017). In the open trial published by Roitman et al. two patients with bipolar disorder that were included in the mixed sample of participants with bipolar- and unipolar-resistant depression turned to hypomania and mania before the 4th week of treatment with creatine monohydrate (3–5 g/day) (Roitman et al. 2007). Regarding the two patients who received creatine that were discontinued from the study due to worsening of depression, it is important to note that they did not present significant mixed features at baseline (total scores on YMRS = 0 and 2).

Side-effects reported during this study were rare, mild and transient, similar to previously published trials that have studied creatine monohydrate as a treatment for various medical, neurological and psychiatric conditions or as an ergogenic (Kim et al. 2011). No subject withdrew from the study due to intolerance to treatment. Adherence rates were high, and these were probably due to the easy once-a-day drug regimen along with the mild side-effects profile of creatine.

There are several limitations in our study that should be considered when interpreting its results. First, it consists in a proof-of-concept trial that did not count on adequate statistical power to consistently detect modest effect sizes, its non-significant findings being subject to type II error. Future proof-of-concept studies on the treatment of bipolar depression with relatively small samples should be designed to increase signal detection, e.g., as sequential parallel comparison trials (Fava 2015). Another limitation is the absence of a treatment arm with a different dose of creatine, which could provide information about the possible efficacy of higher doses, although a recently published 8-week randomized, double-blind, placebo-controlled trial did not find statistically significant differences between adjunctive treatment groups (2, 4 or 10 g/day of creatine monohydrate) in the improvement of depressive symptoms for female adolescents (*N* = 34) with unipolar depression resistant to SSRI (Kondo et al. 2016). Third, our sample was clinically heterogeneous, as we included patients with bipolar disorder type I and II. Fourth, despite both treatment arms being similar in terms of classes of current medication, we did not compare the doses due to the large number of agents. It should be noted that different doses of second-generation antipsychotics may differ in terms of relevant outcomes, such as dropout rates, in subject with bipolar depression (Bartoli et al. 2017) and that there are significant differences in efficacy when single pharmacological agents are compared (Taylor et al. 2014). Fifth, we did not investigate the dietary pattern of the patients.

It remains undetermined whether creatine supplementation for longer periods could have caused more significant effects on depressive symptoms in our sample. Berk et al. added the mitochondrial modulator *N*-acetylcysteine (NAC) to treatment as nusual for bipolar disorder and found significant decreases in depressive symptoms and improved quality of life when compared with placebo, but only after the 20th week of treatment, which suggests that mitochondrial modulators may take a longer time to bring clinical benefits. Berk et al. included patients regardless of mood state, and we can hypothesize that mitochondrial modulators may be more effective as maintenance adjuvant therapy for subsyndromal bipolar depression and not as acute treatment for bipolar-depressed episodes.

Brain bioenergetic status as verified by MRS has been shown to correlate to antidepressant efficacy in clinical trials that studied various substances (Sonawalla et al. 1999; Renshaw et al. 2001; Iosifescu et al. 2008). Pretreatment levels of brain PCr predicted subsequent “responder” vs. “non-responder” *status* in an augmentation trial with triiodothyronine for treatment-resistant unipolar depression with an accuracy of 79% (Iosifescu et al. 2008). In this context, it becomes relevant in future clinical trials in bipolar depression to assess baseline and end point brain and peripheral levels of metabolites involved in the biological effects of creatine to investigate their potential role as mediators, moderators or predictors of response.

Evidence has grown in favor of the selective use of nutritional supplements for the treatment of mood disorders (Sarris et al. 2011, 2016). About 29% of patients with bipolar disorder already take supplements in addition to prescription drugs, and 20% do so in the long run (Bauer et al. 2015). In view of this widespread use of nutritional supplements, scientifically rigorous methods should be used to verify the effectiveness of these supplements and to identify which doses are useful and for whom. It should be noted that effective strategies for the enhancement of mitochondrial functions may require different agents that either modulate multiple mitochondrial targets or increase mitochondrial metabolic activity without concomitant increase in oxidative stress—a strategy that has already been proposed for the treatment of mitochondrial disorders (Rodriguez et al. 2007; Tarnopolsky 2008) and even bipolar depression (Dean et al. 2015) in the form of mitochondrial “cocktails”.

In short, and due to the small sample size, we do not consider that this pilot, proof-of-concept trial is conclusive on the efficacy of creatine for bipolar depression. However, its findings of a superiority of creatine add-on vs. placebo on the rates of remission in the completers and of partial response and remission in the ‘as randomized’ sample suggest that creatine supplementation may have a role in the treatment of this phase of the illness. Further investigation through randomized controlled trials with larger samples should be conducted to verify the efficacy of creatine add-on to standard pharmacotherapy in depressive episodes in bipolar disorder and also verify its utility in subsyndromal depressive symptoms that are highly prevalent and incapacitating throughout the course of the disorder. While extensive published data clearly reinforce the paradigm of mitochondrial and bioenergetic dysfunctionality in the pathophysiology of bipolar disorder, it remains a challenge for translational research to continue investigating new and more effective treatments for this most burdensome phase of the disease.
